# The new histological system for the diagnosis of adrenocortical cancer

**DOI:** 10.3389/fendo.2023.1218686

**Published:** 2023-07-24

**Authors:** Liliya Urusova, Erika Porubayeva, Nano Pachuashvili, Alina Elfimova, Dmitry Beltsevich, Natalia Mokrysheva

**Affiliations:** Department of Fundamental Pathology, Endocrinology Research Centre, Moscow, Russia

**Keywords:** adrenocortical carcinoma, adrenocortical tumor, diagnostic criteria, scoring systems, histological system

## Abstract

**Introduction:**

Adrenocortical cancer (ACC) is a rare malignant tumor that originates in the adrenal cortex. Despite extensive molecular-genetic, pathomorphological, and clinical research, assessing the malignant potential of adrenal neoplasms in clinical practice remains a daunting task in histological diagnosis. Although the Weiss score is the most prevalent method for diagnosing ACC, its limitations necessitate additional algorithms for specific histological variants. Unequal diagnostic value, subjectivity in evaluation, and interpretation challenges contribute to a gray zone where the reliable assessment of a tumor’s malignant potential is unattainable. In this study, we introduce a universal mathematical model for the differential diagnosis of all morphological types of ACC in adults.

**Methods:**

This model was developed by analyzing a retrospective sample of data from 143 patients who underwent histological and immunohistochemical examinations of surgically removed adrenal neoplasms. Statistical analysis was carried out on Python 3.1 in the Google Colab environment. The cutting point was chosen according to Youden’s index. Scikit-learn 1.0.2 was used for building the multidimensional model for Python. Logistical regression analysis was executed with L1-regularization, which is an effective method for extracting the most significant features of the model.

**Results:**

The new system we have developed is a diagnostically meaningful set of indicators that takes into account a smaller number of criteria from the currently used Weiss scale. To validate the obtained model, we divided the initial sample set into training and test sets in a 9:1 ratio, respectively. The diagnostic algorithm is highly accurate [overall accuracy 100% (95% CI: 96%-100%)].

**Discussion:**

Our method involves determining eight diagnostically significant indicators that enable the calculation of ACC development probability using specified formulas. This approach may potentially enhance diagnostic precision and facilitate improved clinical outcomes in ACC management.

## 1 Introduction

Adrenocortical cancer (ACC) is a rare malignant tumor of the adrenal cortex. The reported incidence is 0.5 - 2 cases per 1,000,000 population per year. ACC occurs at any age; however, there is a tendency of increase in the incidence rate in the first and fourth to fifth decades of life, with gender deviations towards the female sex. ACC is mainly sporadic and less often observed as part of hereditary syndromes (1, 2).

There are several histological variants of ACC based on their characteristic cytomorphological features, including conventional, oncocytic, myxoid, and sarcomatoid features (3).

In recent years, ACC has been considered as a heterogeneous group of diseases with various pathomorphological and genomic features, which causes variability in the clinical picture and prognosis for patients (4). An accurate scale for assessing the malignant potential of a tumor is extremely important for deciding on the appointment of adjuvant therapy and determining the features of follow-up of the patient, as well as for predicting immediate and long-term outcomes (5).

Despite the fact that ACC often has a clinically aggressive course, its differential diagnosis from benign adrenocortical adenoma (ACA) can cause difficulties, especially in patients at an early stage with a highly differentiated tumor (6).

In recent decades, the criteria for detecting adrenocortical neoplasms have been improved, which has somewhat simplified the differential diagnosis of ACC and ACA, but diagnostic errors occur in 10-15% of cases (7). Despite the seemingly small percentage of errors, it has a high social significance: it is associated with exhausting regular monitoring and examination, material costs, and is actually unreasonable in the case of overdiagnosis. On the other hand, underdiagnosis does not allow determination of the correct tactics of patient management and prescribing therapy in time, which threatens a fatal outcome.

Currently, there is no single histological criterion that allows the establishment of the malignant nature of an adrenocortical tumor. In the latest clinical guidelines developed jointly by the European Society of Endocrinology (ESE) and the European Network for the Study of Adrenal Tumors (ENSAT), the Weiss system is recommended as the preferred system for determining the malignant potential of ACC in adults and is by far the most widely used system (8).

The aim of this work was to develop a mathematical model with high sensitivity and specificity for determining the malignant potential of adrenocortical tumors, which can be used to diagnose all morphological variants of ACC in adults.

## 2 Materials and methods

### 2.1 Patients and samples

The study included tumor tissue samples from patients with adrenal neoplasms who were treated at the Endocrinology Research Centre. All patients underwent adrenalectomy in the period from 2005 to 2022. Patients under the age of 18 at the time of surgical treatment were not included in the study. A total of 143 cases of adrenal tumors were selected for the study.

### 2.2 Morphological examination

Tumor tissue samples obtained during surgical treatment of patients at the Endocrinology Research Center were fixed in 10% buffered formalin, processed in the histological wiring system of a Leica ASP200, and poured into paraffin. Further, paraffin sections with a thickness of 3 µm were made from the paraffin-embedded tumor tissue samples on a microtome, and applied to slides treated with poly(l-lysine). Then the slides were stained with hematoxylin and eosin according to the standard procedure.

All tumor tissue samples were verified in accordance with the 2022 WHO classification of adrenal cortical tumors.

### 2.3 Immunohistochemistry imaging

Immunohistochemical analysis of the tumor tissue sections was carried out according to the standard technique with a peroxidase detection system with DAB on an automatic Leica BOND III IHC staining system using Leica reagents. Ki-67 (ACK02, Leica) and PHH3 (PA0817, Leica) antibodies were used. All histological slides were scanned using a Leica Aperio AT2 system at 20x magnification for further analysis. The Ki-67 proliferation index was calculated according to the formula: number of stained nuclei/2,000 cells x 100%, the area of greatest Ki-67 positivity (hot spot). Mitotic count was carried out in ten fields of view with 400x magnification. The most representative fields of view were selected.

### 2.4 Statistical analysis

Statistical analysis was carried out on Python 3.1 in the Google Colab environment. The cutting point was chosen according to Youden’s index. Scikit-learn 1.0.2 was used for building the multidimensional model for Python. Logistical regression analysis was executed with L_1_-regularization, which is an effective method for extracting the most significant features of the model.

Model equation:


$$p=1/\left(1+e^{-z}\right)$$

$$Z=\beta_{0}+\beta_{1}*X_{1}\;+\;\beta_{2}*X_{2}+\ldots +\;\beta_{i}*X_{i}$$

Z – dependent binary target,

$X_{1}$ , …, $\;X_{i}$ – independent features,

$\beta_{0}$ , …, $\beta_{i}$ – coefficients.

The loss function for the logistical regression model:

Loss_Function = logloss + λ_1_|| β ||_1_, where

logloss – function logloss;

β – weight of parameters;

λ_1_ – L_1_-regularization coefficient;

|| β ||_1_ – L_1_ – weight norm;

L1-norm:


$$∥\text{β}{\|}_{1}=\sum_{i=1}^{n}\left|{\text{β}}_{i}\right|$$

To validate the obtained model, we divided the initial sample set into training and test sets in a 9:1 ratio, respectively. The operational characteristics used were: diagnostic sensitivity (DS), diagnostic specificity (DSp), positive predictive value of result (PPV), and negative predictive value of result (NPV). 

## 3 Results

### 3.1 Design of the system for the risk stratification of adrenocortical tumors

The sample size was 143 patients, who were divided into training (n=128) and testing (n=15) groups. The features were analyzed in terms of their informativeness in relation to the diagnosis of ACC. The first stage executed was to assess whether tumor size > 10cm and/or tumor size >200g. If the tumor corresponded to these criteria, the histological diagnosis was consistent with ACC in 100% of cases.

**Figure 1:**
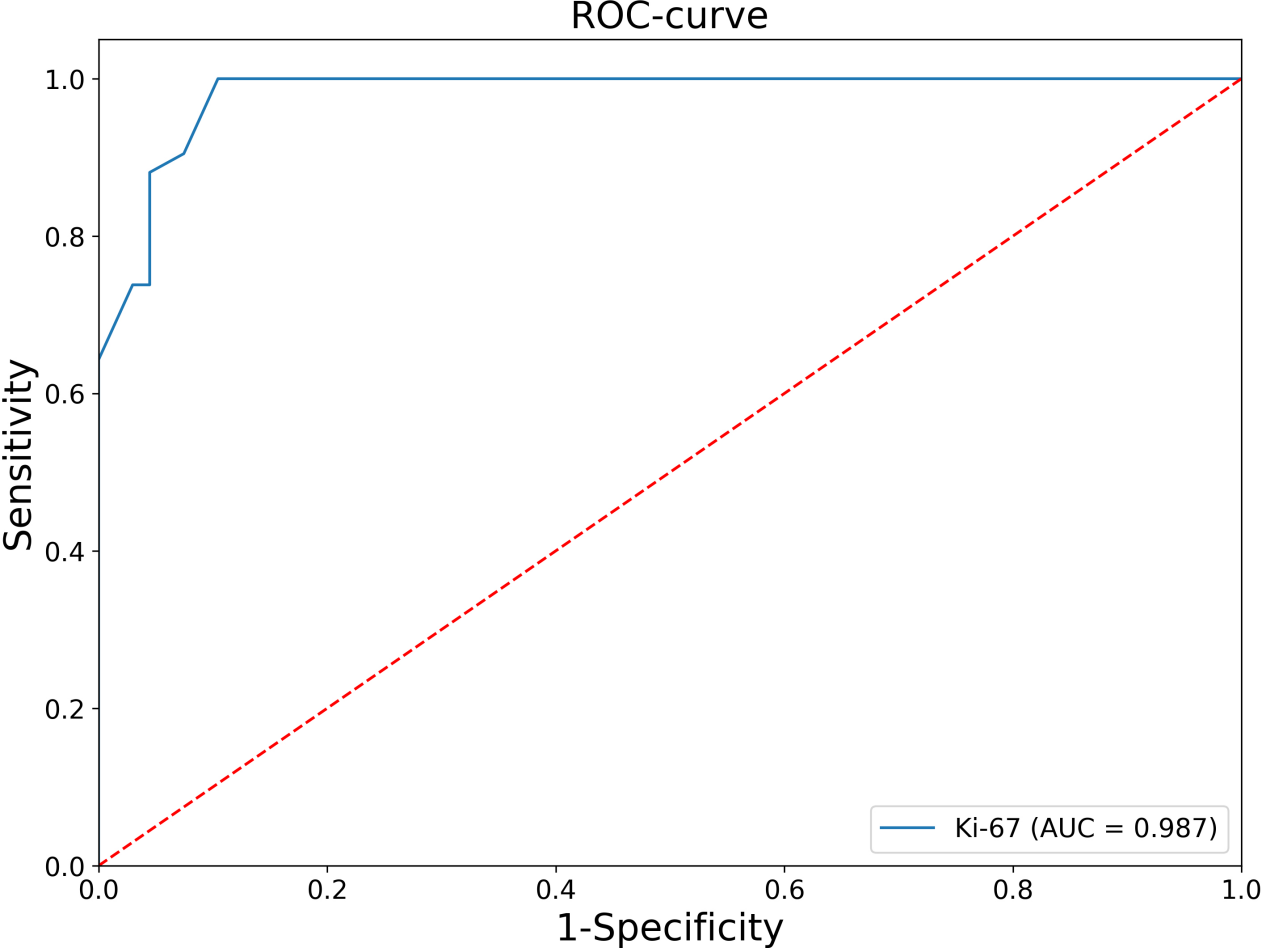
ROC analysis of the Ki-67 levels in relation to the diagnosis of ACC.

At the second stage, patients with negative values of this criteria were analyzed, that is, tumor size ≤10 cm and tumor weight ≤ 200 g, and Receiver Operating Characteristic (ROC) analysis of the Ki-67 index was performed (**Figure 1**).

The Area under the ROC Curve (AUC) was 0.987 (95% CI: 0.969-1.000), which corresponds to a high diagnostic efficiency for this feature. According to the Youden’s index, a Ki-67 cut-off point was chosen equal to 5%. The confusion matrix of this point is presented in **Table 1**.

**Table 1:** Confusion matrix for diagnostics of ACC using a Ki-67 cut-off point of 5% (n= 95).

	ACC	ACA/tumor of uncertain malignant potential
Ki-67 ≥ 5%	36	6
Ki-67< 5%	0	53

**Figure 2:**
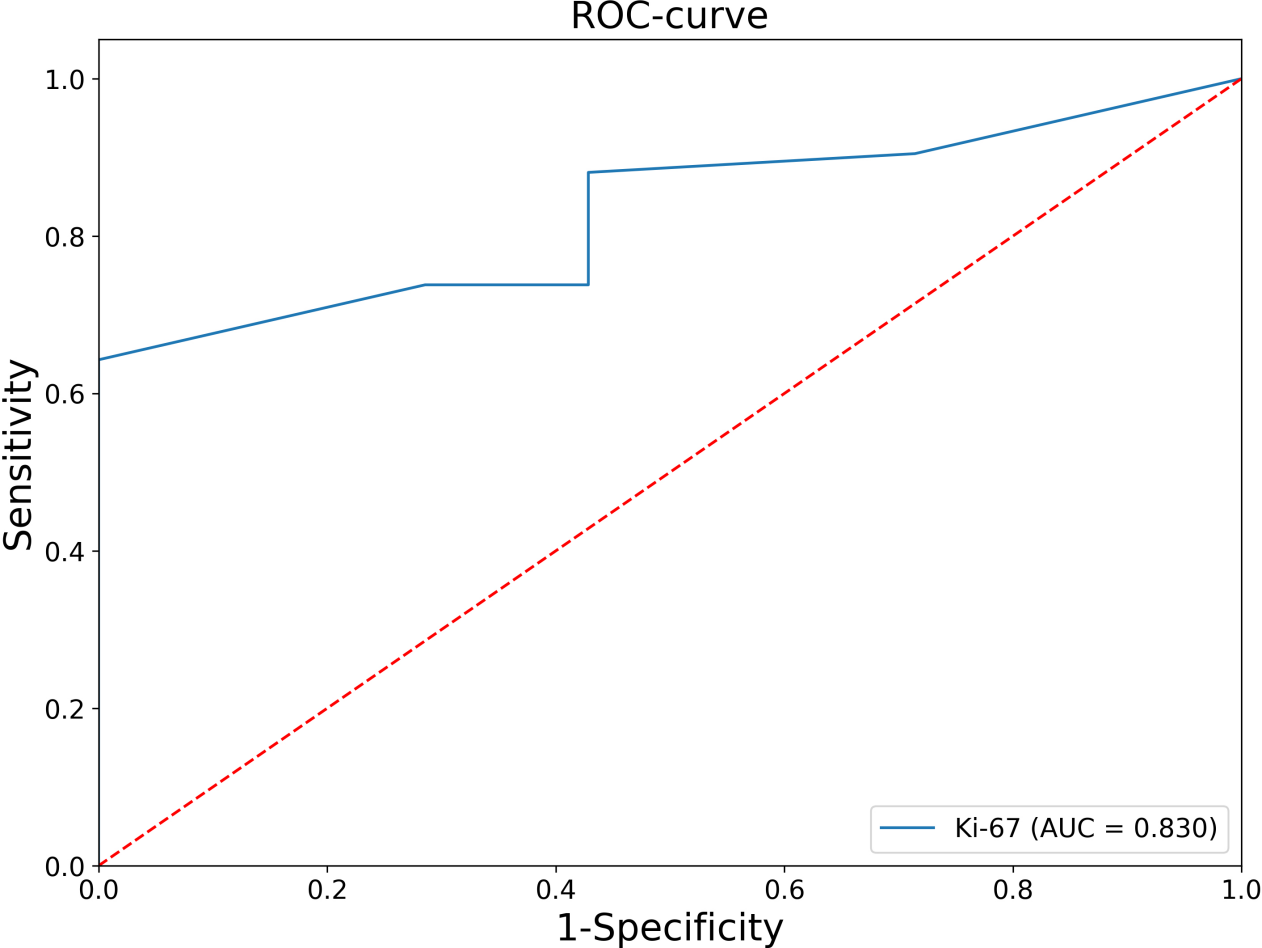
ROC analysis of the Ki-67 levels for patients whose Ki-67 level was greater or equal to 5%.

Thus, in all patients with Ki-67<5%, the histological diagnosis is ACA. At the next stage, an ROC-analysis of the Ki-67 was also performed for patients whose Ki-67 level was greater or equal to 5% (**Figure 2**).

The AUC was 0.830 (95% CI: 0.696-0.964), which corresponds to a satisfactory diagnostic efficiency for this feature. According to Youden’s index, a Ki-67 cut-off point was chosen equal to 11%. The classification matrix for a given point is presented in **Table 2**.

**Table 2:** Classification matrix for diagnostics of ACC using a Ki-67 cut-off point equal to 11% (n= 42).

	ACC	ACA/tumor of uncertain malignant potential
Ki-67 ≥ 11%	23	0
Ki-67< 11%	13	6

The final stage was to create a mathematical model for the purpose of differential diagnosis of ACC and ACA/neoplasms of undetermined malignant potential for patients with a Ki-67 value in the range of 5% to 10% inclusive. Logistic regression analysis with L1-regularization was used. The response was histological diagnosis. The set of predictors used were:

1. Size of tumor (max) – numeric parameter;
2. Phosphohistone H3 (PHH3) – numeric parameter;
3. Ki-67 – numeric parameter;
4. Mitoses – binary parameter in format (1/0, which corresponds yes/no);
5. Nuclear grade – binary parameter in format (1/0, which corresponds yes/no);
6. Atypical mitoses – binary parameter in format (1/0, which corresponds yes/no);
7. Invasion of capsule – binary parameter in format (1/0, which corresponds yes/no);
8. Diffuse architecture of tumor – binary parameter in format (1/0, which corresponds yes/no);
9. Invasion of venous structures – binary parameter in format (1/0, which corresponds yes/no);
10. Necroses – binary parameter in format (1/0, which corresponds yes/no);
11. Eosinophilic cells – binary parameter in format (1/0, which corresponds yes/no).

As a result, the following regression model was obtained:


Z=−0.018∗Хsize+0.278∗Хmitoses −0.261∗Хnuclear grade+0.297∗Хpathological mitoses+0.816∗Хinvasion of capsule+0.565∗Хnecroses

$$p=1/\left(1+e^{-z}\right)$$

The weight of the remaining features of the model was equal to 0, that is, the remaining features are not taken into account when making a diagnosis. If the p-value is ≥0.5, then histological diagnosis will correspond to ACC; if the p-value is<0.5, it corresponds to ACA. The classification matrix is represented in **Table 3**.

**Table 3:** Classification matrix for diagnostics of ACC using a logistic regression model (n= 19).

	ACC	ACA/tumor of uncertain malignant potential
Result of the model – ACC	13	3
Result of the model – ACA	0	3

### 3.2 The stages of diagnostics

**Figure 3:**
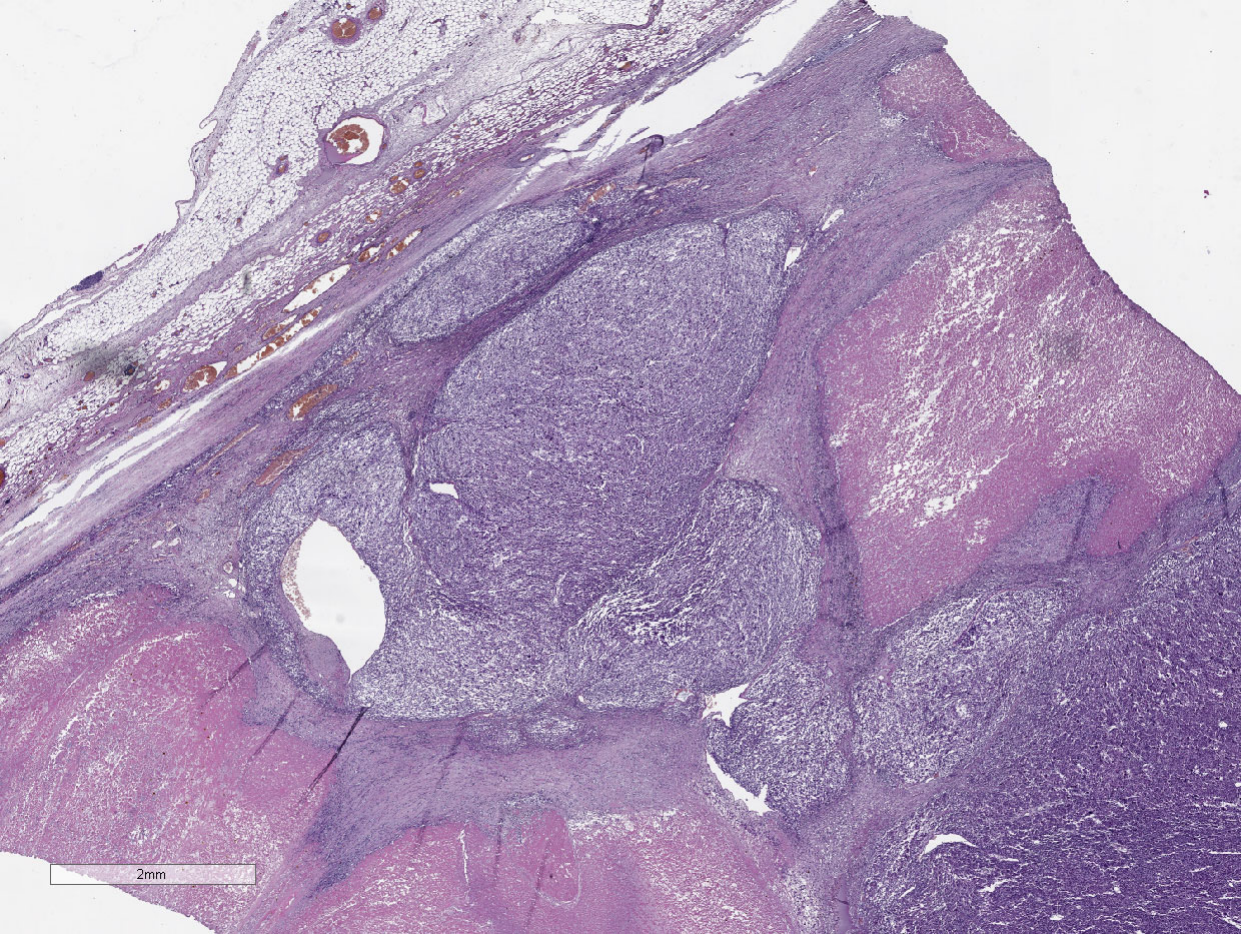
ACC, the area of necrosis. Hematoxylin and eosin stain. Scale х100.

**Figure 4:**
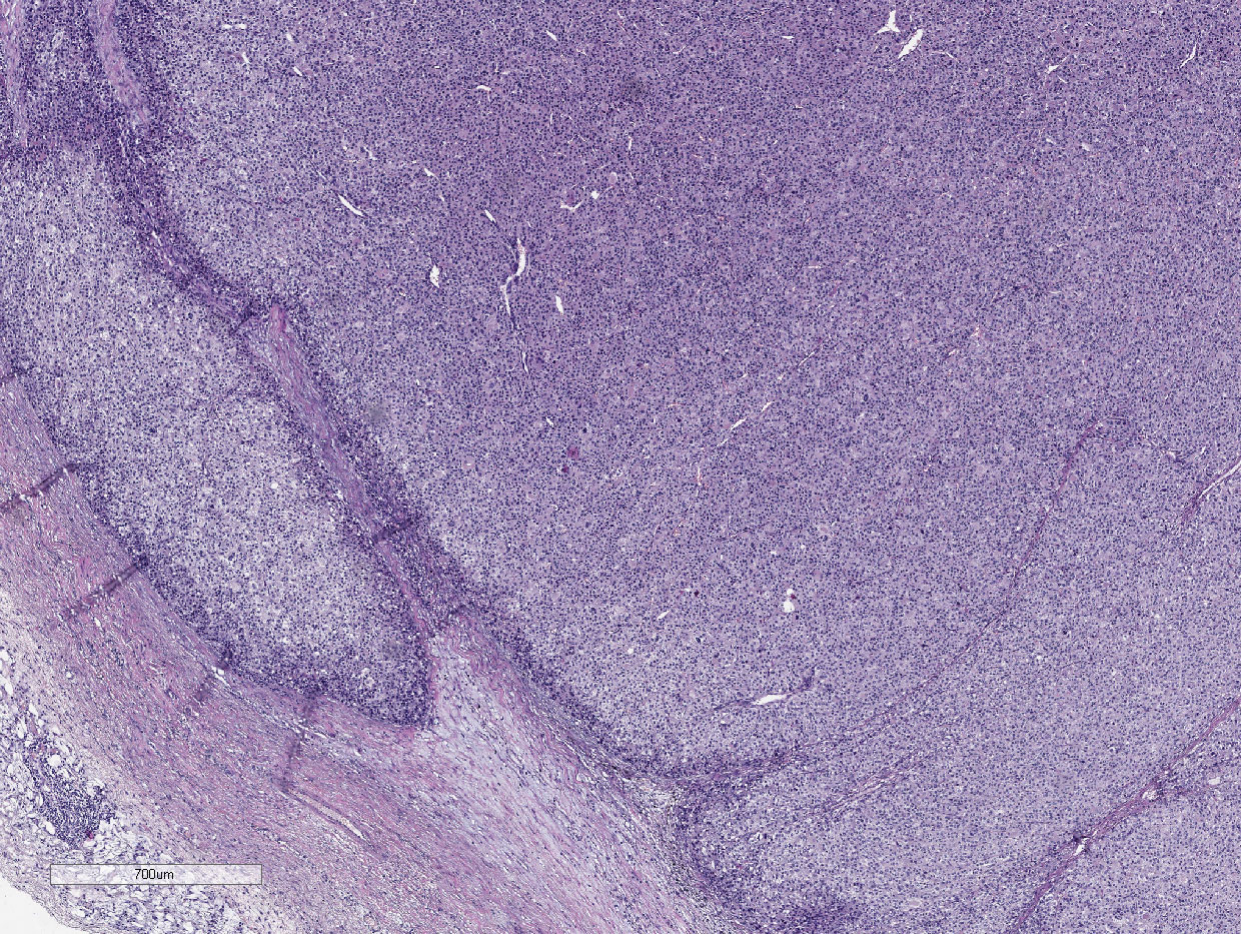
ACC, invasion of capsule. Hematoxylin and eosin stain. Scale х100.

**Figure 5:**
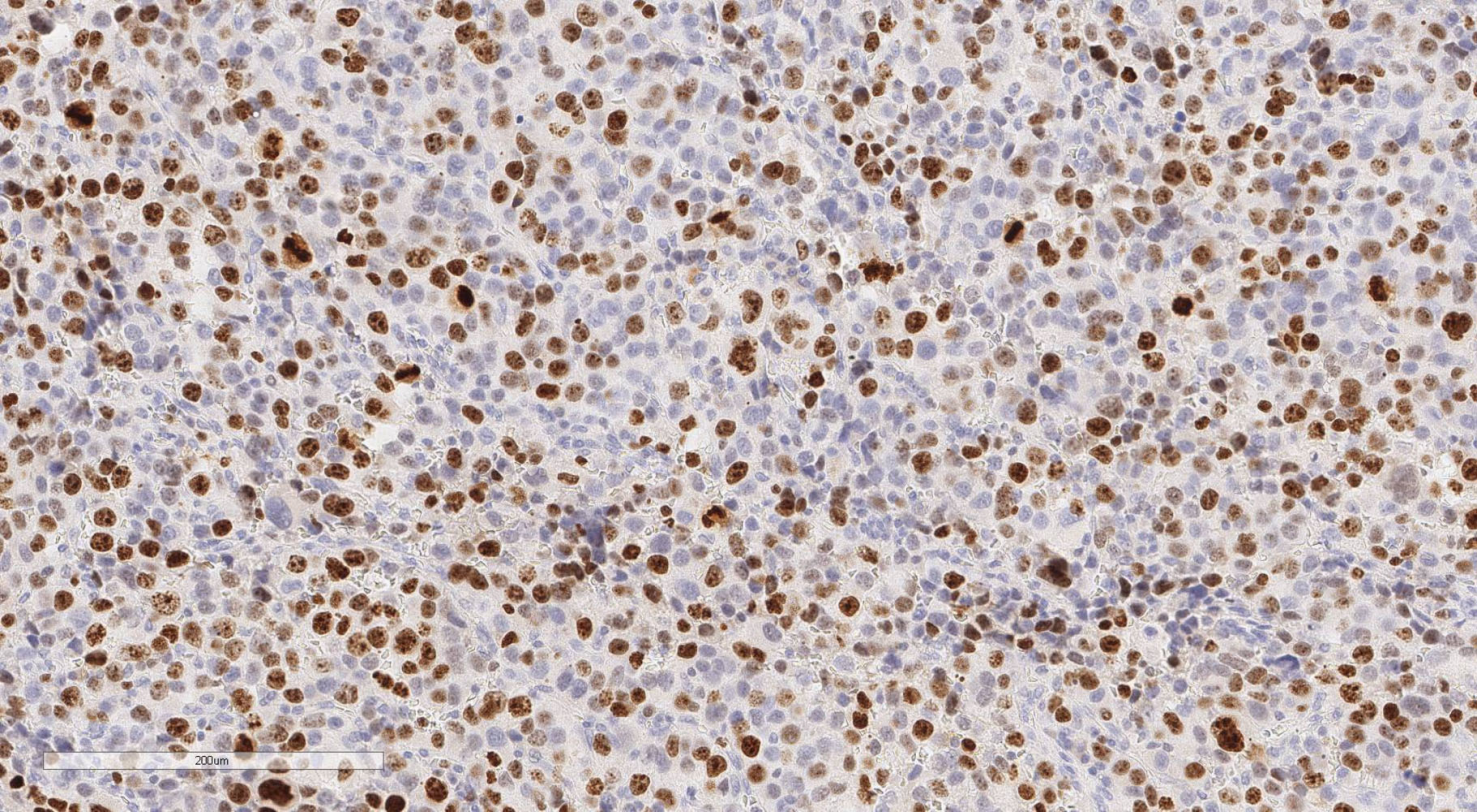
ACC, immunohistochemical stain with Ki-67 antibody. Scale х100.

Thus, eight parameters are used in ACC diagnostics (**Figures 3**–**5**):

1. Tumor size
2. Tumor weight
3. Ki-67
4. Mitoses
5. Nuclear grade
6. Atypical mitoses
7. Invasion of capsule
8. Necroses

**Figure 6:**
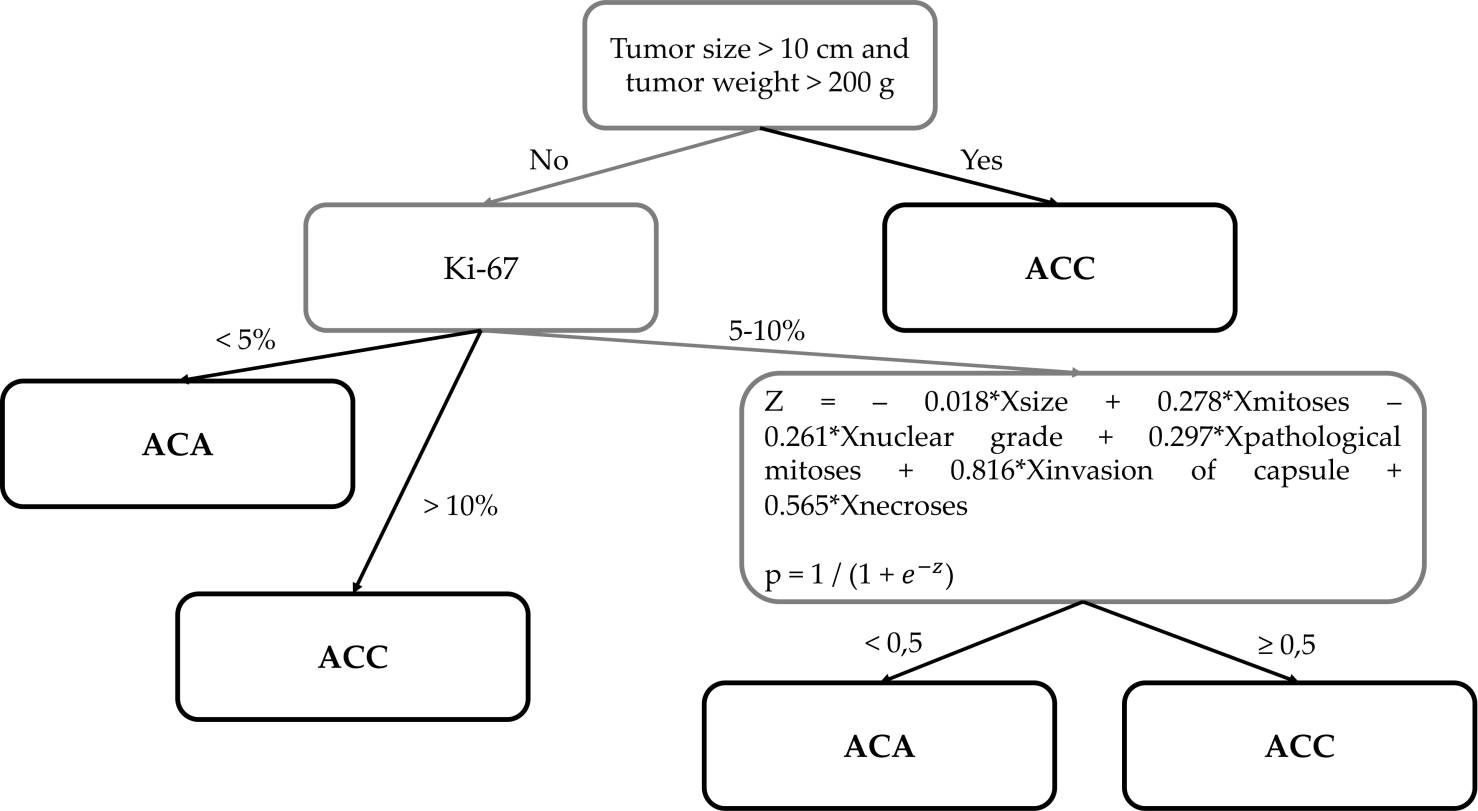
The new histological system for the diagnosis of adrenocortical cancer of the Moscow Endocrinology Research Centre.


Diagnostics are carried out in three stages (**Figure 6**):

- 1. Size >10 сm and/or weight >200 g → Diagnosis: ACC
- 2. Ki-67<5% → Diagnosis: ACA
- Ki-67 ≥11% → Diagnosis: ACC
- 3. Ki-67 = 5-10%

Z = – 0.018∗Хsize+0.278∗Хmitoses −0.261∗Хnuclear grade+0.297∗Хpathological mitoses+0.816∗Хinvasion of capsule+0.565∗Хnecroses

$$p=1/\left(1+e^{-z}\right)$$

р ≥0.5 → Diagnosis: ACC

p<0.5 → Diagnosis: ACA

The final classification matrix is presented in **Table 4**.

**Table 4:** Final classification matrix for diagnostics of ACC (n= 143).

Training sample	ACC	ACA/tumor of uncertain malignant potential
Result of the diagnostics – ACC	68	3
Result of the diagnostics – ACA	0	57
Testing sample		
Result of the diagnostics – ACC	8	0
Result of the diagnostics – ACA	0	7

The operational characteristics of the test set were:

DS = 100%

DSp= 100%

PPV =100%

NPV = 100%

There were three false-positive results. The model classified these three cases with tumor of uncertain malignant potential as ACC. However, metastases were developed in these three patients, which allowed them to be attributed to the ACC group. Thus, the model classified all patients correctly.

### 3.3 Examples

**Example 1**. Patient L., 66 years old. She complained of episodes of increased blood pressure up to 200/120 mm Hg, general weakness, and increased fatigue. Ultrasound examination revealed a mass in the region of the left adrenal gland.

Multispiral computed tomography (MSCT) revealed a solid lesion of the left adrenal gland with a maximum diameter of 37 mm and a native density of 25-35 Hounsfield Units (HU). Aldosterone – 120 pg/ml, renin – 0.22 ng/mL/hr (2.79 – 61.83), ARR – 545 (<300). In a 24-hour urine test, methylated catecholamines were normal. During the examination of dynamics, aldosterone – 218 pg/ml (<199), renin – 1.566 ng/mL/hr (2.79 – 61.83), and ARR – 545 (<100). In an overnight cortisol suppression test with 1 mg dexamethasone – 2 μg/dL, ACTH – 8.9 pg/ml (7.2 – 63.3).

18F-FDG PET/CT revealed hyperfixation of the radiopharmaceutical in the formation of the left adrenal gland, SUV max 21.33. A preliminary diagnosis was made as a tumor of uncertain malignant potential.

The patient was admitted to the surgical department, where a planned endoscopic left-sided adrenalectomy was performed. The results of the pathoanatomical study are presented in **Table 5**.

**Table 5:** Results of a pathoanatomical study of patients with adrenal tumors.

	1	2	3	4
Tumor size, cm	3.7	6.7	4.0	5.0
Tumor weight, g	40	70	30	40
Ki-67, %	10	15	10	6
Mitoses	No	Yes	No	No
Nuclear grade	No	No	No	Yes
Atypical mitoses	No	Yes	Yes	No
Capsular invasion	No	Yes	Yes	No
Necrosis	Yes	Yes	No	No
Diffuse architecture	No	No	Yes	No
Sinusoidal invasion	No	No	No	No
Vascular invasion	No	No	No	No
Eosinophilic cells	No	Yes	Yes	No
Weiss/Lin— Weiss—Bisceglia	1 minor criteria	5 points	1 major and 1 minor criteria	1 point
Diagnosis according to the Weiss/Lin—Weiss—Bisceglia system	Tumor of uncertain malignant potential	ACC, myxoid variant	ACC, oncocytic variant	ACA
Diagnosis according to the system	ACC	ACC	ACC	ACA

In January 2023, the spread of the tumor process was diagnosed as locoregional recurrence. Relapse-free survival was 12 months.

**Example 2**. Patient P., 58 years old. She complained of episodes of increased blood pressure up to 180/120 mm Hg. According to the patient, in 2012, a formation in the left adrenal gland with dimensions of 27x15x17 mm was diagnosed for the first time.

According to MSCT from 2018, a formation with dimensions of 67x46x46 mm, ovoid in shape, and with a native density of 39-57 HU was found in the left adrenal gland.

According to the results of examination, the tumor produces hormones (hypercorticism): cortisol – 375.7 nmol/l, ACTH – 4.1 pmol/l (6 – 58), aldosterone – 130.7 pg/ml (<199), and renin – 3.9 ulU/ml (2.79 – 61.83).

Given the high malignant potential of the formation in the projection of the right adrenal gland, planned surgical treatment was performed in the volume of left-sided adrenalectomy with a tumor. The results of the pathoanatomical study are presented in **Table 5**.

At the moment, the patient is alive with no signs of disease progression, is disease-free, and overall survival is 15 months.

**Example 3**. Patient S., 53 years old. During examination in May 2015, according to ultrasound data, a mass of about 44 mm in size was found in the right adrenal gland. During a second examination in May 2016, the mass was measured to be about 41 mm in size.

MSCT revealed a lesion of the right adrenal gland of 42х33х25 mm with a native density of 38-56 HU. According to the results of the survey, no data in favor of hormonal activity was obtained.

The patient was provisionally diagnosed with a tumor with uncertain malignant potential. Surgical treatment was performed in in the volume of right-sided adrenalectomy with a tumor. The results of the pathoanatomical study are presented in **Table 5**.

At the time of the survey, the patient’s relapse-free and overall survival time were 13 and 37 months, respectively. However, a systemic relapse was later established, and the patient died.

**Example 4**. Patient Y., 49 years old. Complained of increased blood pressure to 280/160 mm Hg., against a background of constant multicomponent antihypertensive therapy, back pain with irradiation to the lower limbs, severe weakness, and a weight gain of 12 kg over the past 3 months.

According to the results of examination: aldosterone – 644 pg/ml (<199), renin – 1.67 ng/mL/hr (2.79 – 61.83), and cortisol – 1351.89 nmol/l, ACTH – 24.8 pmol/L (6 – 58).

According to MSCT, a formation with the dimensions 53x27x21mm with a native density of 32-52 HU was found in the left adrenal gland.

Performed surgical treatment in the volume of of left-sided adrenalectomy with a tumor. The results of the pathoanatomical study are presented in **Table 5**.

## 4 Discussion

In 1984, Louis Weiss proposed a histopathologic classification system for adrenocortical tumors, based on nine criteria (**Table 6**). The presence of more than three or more features is consistent with adrenocortical carcinoma (9, 10).

**Table 6:** Systems for assessing the malignant potential of adrenocortical tumors.

Weiss System		Lin— Weiss—Bisceglia System		Helsinki Score	
Parameter	Score	Criteria	Parameter	Parameter	Score
Nuclear grade* ≥ 3		Criteria for inclusion in a group of oncocytic tumors	Eosinophilic cytoplasm	> 5 mitoses/50 HPF	3
	1		High nuclear grade	Necrosis	5
			Diffuse architecture	Proliferative Index (Ki-67 IHC)	Numeric value
> 5 mitoses/50 HPF 1	1	Major criteria	> 5 mitoses/50 HPF 1		
Atypical mitoses	1		Atypical mitoses		
Clear cells ≤ 25%	1		Venous invasion		
Diffuse architecture > 33%	1	Minor criteria	Large size (> 10 cm) or weight (> 200 g)		
Confluent necrosis	1		Necrosis		
Vascular invasion	1		Sinusoidal invasion		
Sinusoidal invasion	1		Capsular invasion		
Capsular invasion	1				
**Interpretation**		**Interpretation**		**Interpretation**	
Final score ≥ 3	ACC	One more major criterion	ACC	Final score = 0-8.5	ACA
		One more minor criteria	Tumor of uncertain malignant potential	Final score > 8.5	ACC
		The absence of all major and minor criteria	Benign tumor	Final score > 17	Unfavorable prognosis

* The nuclear grade is assessed according to the Fuhrman criteria as follows: grade 1: small and round nuclei, non-visible nucleoli; grade 2: slightly larger and irregularly shaped nuclei, nucleoli visible with a high-magnification lens; grade 3: irregular and enlarged nuclei, nucleoli visible with a low magnification lens; grade 4: bizarre and extremely irregular nuclei, including monstrous cells. ACA, adrenal cortical adenoma; HPF, high-power fields (equivalent to almost 10 mm2).

In most adrenocortical adenomas, Weiss parameters are not detected, whereas in most cases of ACC (62% in a series of 201 cases in Turin, Italy), 6 or more points are noted, and their malignant nature is beyond doubt (11). However, cases of neoplasms of the adrenal cortex, in which only 1-2 points are detected according to Weiss parameters (approximately 10% of cases), are neoplasms with uncertain malignant potential. At this stage, the determination of their clinical course is an unsolved problem, and calls into question the reliability of the Weiss system and emphasizes the necessity of a more unambiguous scale for the verification of ACC.

In most of the “borderline” cases of Weiss score, the following parameters are most often identified: nuclear grade, diffuse architecture, or sinusoidal invasion. At the same time, it is these parameters that usually have the lowest specificity relative to the correlation with malignant behavior. Other parameters associated with malignant potential, such as a high mitotic rate, atypical mitoses, necrosis, and venous and capsular invasion, are most often not detected in isolation, but are combined with other parameters, thus demonstrating higher scores on the Weiss scale.

The reproducibility of the scale between different pathologists is another factor affecting the diagnostic effectiveness of the Weiss score, since the determination of some parameters, namely, diffuse architecture and sinusoidal and vascular invasion, are very subjective (12).

It is important to note that some parameters of the Weiss system (for example, the absence of diffuse architecture, nuclear atypia, or the invasion of lymphatic vessels) are difficult to assess in the case of a myxoid histological subtypes, since a large amount of myxoid substance makes it difficult to assess the stromal elements of the tumor (13). This may lead to the underestimation of the malignant potential of neoplasms.

Moreover, the use of the Weiss score for oncocytic tumors is not recommended, since oncocytic neoplasms consist of cells with eosinophilic cytoplasm, high nuclear grade, and diffuse architecture, which will inevitably lead to an erroneous diagnosis of ACC, which in turn contradicts their more frequent benign biological behavior (14). Therefore, the Lin–Weiss–Bisceglia system was developed to assess the malignancy of oncocytic variants of adrenal cortical tumors (15).

The Lin–Weiss–Bisceglia system includes “major criteria” and “minor criteria”. The diagnosis of ACC is established in the presence of at least one of the “major criteria”. In case of the presence of at least one “minor criterion” the tumour is considered as an oncocytic adrenal cortical neoplasm with uncertain malignant potential. If there are no both “major criteria” and “minor criteria”, an oncocytic tumor is regarded as benign.

At the same time, for the application of this system, it is important that oncocytic adrenal cortical neoplasms are extensively sampled to be certain that they do fit into the category of pure oncocytic tumors. This means that greater than 90% of the tumor must be oncocytic (16). If it is not a pure oncocytic adrenal cortical neoplasm, then the system applied to conventional adrenal cortical carcinomas should be used.

The updated classification of the 2022 World Health Organization (WHO) also suggests using other multiparametric diagnostic algorithms for morphological assessment of neoplasms of the adrenal cortex in adults. These include the reticulin algorithm (17) and the Helsinki score (18), which, according to the literature, can be used for conventional, oncocytic, and myxoid variants of neoplasms.

The reticulin algorithm is based on the detection of changes in the normal structure of the reticulin network (Gordon and Sweet’s silver histochemical stain) in combination with one of the following parameters: the presence of mitotic rate more than 5 per 10 mm^2^ (50 high-power fields), tumor necrosis, or vascular invasion. However, this method has not found wide application in pathomorphological practice due to its technical complexity.

The Helsinki Score proposes to summarize the numeric value of the Ki-67 labeling index and the scores assigned to increased mitotic rate (a score of 3 is given for a mitotic rate greater than 5 mitoses per 10 mm^2^) and tumor necrosis (5 points). A Helsinki score of > 8.5 is a diagnostic sign of ACC, and a score of > 17 makes it possible to regard the tumor as prognostically unfavorable.

Among all the proposed assessment systems, according to the latest data, the Helsinki score is the most reliable (19). However, it should be borne in mind that the assessment of mitoses in the cells of myxoid and oncocytic variants of ACС may be difficult, which may also lead to underestimation of the malignant potential of the tumor. At the same time, the clinical course of the myxoid variant in some cases is much more aggressive in comparison with other ACС variants. In this regard, it is necessary to be wary of the malignant nature of myxoid tumors.

Thus, the Weiss system, which is a standard of diagnosis, cannot be regarded as a universal system, which significantly complicates the diagnostic process because additional algorithms are required for specific histological variants. The system we have developed is a diagnostically meaningful set of indicators that takes into account a smaller number of criteria from the currently used Weiss scale. The diagnostic algorithm is highly accurate [overall accuracy 100% (95% CI: 96%-100%)]. Its main benefit is its universal applicability to all morphological variants of ACC in adult patients, which distinguishes this approach from the current known methods. Importantly, it is also applicable in standard conditions of medical pathomorphological laboratories, i.e., it does not require any parameters that are not assessed in routine examination of histological material.

The developed method includes evaluation of a set of eight diagnostically relevant parameters: tumor size (cm), tumor weight (g), Ki-67 index (%), presence/absence of mitoses, nuclear polymorphism, abnormal mitoses, invasion into the capsule, and necroses. Based on the data obtained, the probability of ACC development is calculated according to formulas in three steps.

It is worth noting that three false-positive results (classified as ACC) were detected, but these cases were diagnosed as tumors of uncertain malignant potential rather than a confirmed ACC. Therefore, it is incorrect to consider this a diagnostic error, as this group of patients required dynamic monitoring to determine their final diagnosis. Moreover, one of these three cases has a locoregional recurrence at the time of writing (January 2023), which confirms the presumed malignant course of the disease according to the new reclassification system.

## 5 Conclusions

Thus, for patients with a verified histological diagnosis, the developed mathematical model showed 100% accuracy in the training and test samples. The application of the new model could solve the problem of subjectivity and complexity in interpretation of certain criteria of the diagnostic algorithms currently used in clinical practice. Furthermore, the new model is unique in that, unlike others, it allows verification of all morphological variants of ACC. Thus, the results of our study suggest that the application of the new system will improve and standardize the differential diagnosis of adrenocortical tumors.

## Data availability statement

The original contributions presented in the study are included in the article/supplementary material. Further inquiries can be directed to the corresponding author.

## Ethics statement

The studies involving human participants were reviewed and approved by Institutional Ethics Committee of Endocrinology Research Centre (protocol code 10, 26.05.2020). The patients/participants provided their written informed consent to participate in this study.

## Author contributions

LU, AE, DB, and NM designed the research. LU and AE performed the experiments. LU and AE analyzed the data. LU, EP, NP, and AE discussed the data. LU, EP, and NP wrote the paper. All authors contributed to the article and approved the submitted version.
